# Preparation and Closed-Loop Recycling of Ultra-High-Filled Wood Flour/Dynamic Polyurethane Composites

**DOI:** 10.3390/polym15061418

**Published:** 2023-03-13

**Authors:** Shiyu Guo, Huanbo Wang, Yue Liu, Yuan Fu, Xuefeng Zhang, Bin Qi, Tian Liu

**Affiliations:** Key Laboratory of Bio-Based Material Science & Technology, Northeast Forestry University, Ministry of Education, 26 Hexing Road, Harbin 150040, China

**Keywords:** ultra-high biomass, composites, closed-loop recycling, dynamic phenol–carbamate bonds, in situ polymerization

## Abstract

The development of biomass-based composites has greatly reduced the daily consumption of plastics. However, these materials are rarely recyclable, thus, posing a severe threat to the environment. Herein, we designed and prepared novel composite materials with ultra-high biomass (i.e., wood flour) filling capacity and good closed-loop recycling properties. The dynamic polyurethane polymer was polymerized in situ on the surface of wood fiber, and then they were hot-pressed into composites. Fourier-transform infrared (FTIR), scanning electron microscopy (SEM), and dynamic thermomechanical analysis (DMA) measurements reveal good compatibility between the polyurethane and wood flour in the composites when the wood flour content is ≤80 wt%. The maximum tensile and bending strength of the composite are 37 and 33 MPa when the wood flour content is 80%. The higher wood flour content results in higher thermal expansion stability and creep resistance in the composites. Moreover, the thermal debonding of dynamic phenol–carbamate bonds facilitates the composites to undergo physical and chemical cycling. The recycled and remolded composites exhibit good mechanical property recovery rates and retain the chemical structures of the original composites.

## 1. Introduction

Composites prepared from plant fibers, such as wood flour and petroleum-based plastics, are often non-biodegradable and have limitations in processing methods, which prevent carbon neutrality and a circular economy from being achieved [1]. Moreover, the excessive use of petroleum-based plastics in composite materials has caused severe environmental pollution problems and posed a long-term threat to human health [2]. Some researchers tried to prepare biocomposites using biodegradable plastics. However, the biocomposites have a low biomass percentage, thus, limiting the use of biomass [3,4,5]. Novel, environmentally friendly, and renewable biomass-based composite materials are urgently needed in modern society.

Conventional wood composites, such as plywood, fiberboard, and particleboard, are prepared from thermosetting resins and different sizes of wood fibers that are mostly not recyclable. Wood–plastic composites (WPCs) prepared by filling a continuous phase (thermoplastic polymer) with rigid particles (biomass fibers) are thermoplastic recyclables [6]. However, WPCs with higher biomass fiber fillings suffer from the following problems: (i) the fiber agglomeration caused by the lack of polymer matrix [7]; (ii) poor compatibility between wood flour and non-polar polyolefin-based plastics, which leads to interfacial defects in the composites [8]; (iii) increased viscosity and decreased flowability of the composite melts, leading to molding difficulties [9] and low productivity and quality of the products. In addition, melt processing is energy intensive [10] because melt-processed biocomposites undergo heating and cooling several times to produce the final product. In contrast, the processing of biocomposites via in situ polymerization uses lower cumulative energy [11]. The use of an in situ pre-polymerization processing method on a wood fiber network followed by hot pressing is a viable means of achieving high-fiber-content biomass composites. Berglund et al. [12] designed and investigated the preparation of CNF/bio-epoxy biocomposites via in situ polymerization of bio-based epoxy resins on porous nanocellulose networks. They prepared a biomass composite with high CNF content and nanofibers uniformly dispersed in the bio-epoxy thermoset matrix. However, they adopted the solvolysis recycling method to transform waste thermosets into soluble monomers or oligomers. In this process, wastes are usually decomposed in acidic and alkaline solvents, which can lead to higher energy consumption and health and safety risks [13].

Recently, the closed-loop chemical recycling strategy has attracted considerable scholarly attention [13,14,15,16]. Closed-loop recyclability can be achieved by incorporating cleavable or dynamic chemical bonds into a thermoset network to achieve controlled degradation of conventional thermosets under certain conditions [13]. Polymer networks with dynamic covalent bonds that can undergo exchange reactions can relax stresses and reconfigure the network topology when stimulated under external conditions, such as temperature, allowing these polymers to be thermally processed and recycled [17,18,19,20,21]. The replacement of traditional petroleum-based thermosetting polymers with dynamic covalent bonding polymers is an ideal solution for the non-degradable recycling of biomass composites. Dynamic covalent-bond-containing polymers have better thermal stability and chemical stability than thermoplastic polymers. In addition, dynamic covalent polymers have significant advantages of reprocessing and recycling over thermosetting polymers [18]. Dynamic covalent bonds, such as disulfide bonds [22], diselenide bonds [23,24], boron esters bonds [25,26], hindered urea bonds [27,28], and Diels–Alder bonds, have attracted great attention [29,30]. Moreover, the phenol–carbamate bond is of interest to researchers because of its excellent kinetic properties and suitable dissociation temperature [31,32,33,34]. The introduction of dynamic covalent chemistry gives woody biomass materials new functions such as reprocessability and recycling, but existing dynamic polymer/biomass composites usually have a low biomass fraction and complex synthesis processes.

In this study, we designed a novel dynamic polyurethane polymer containing phenol–carbamate bonds to prepare polymeric biomass composites via in situ polymerization on the surface of wood fibers followed by hot compression molding. The properties of the ultra-high-filled wood flour/dynamic polyurethane composites (UWPU) were measured by FTIR, SEM, DMA, TMA, and creep test. Moreover, the composite materials (containing 60–90 wt% biomass) with remolding, complete recycling, and good mechanical properties prepared through this method provide scalable new ideas for developing new biomass composites.

## 2. Materials and Methods

### 2.1. Materials

Poplar wood flour was purchased from Harbin, China. The particle size of the poplar wood flour was 40 mesh. The wood flour was dried at 105 °C for 8 h in a vacuum oven before use. Isophorone diisocyanate (IPDI) was purchased from Shanghai Macklin Biochemical Co., Ltd., Shanghai, China. Poly ethylene glycol (PEG, Mn 600) and N, N-dimethylformamide (DMF) were purchased from Shanghai Aladdin Biochemical Technology Co., Ltd., Shanghai, China. Tannic acid (TA, purity~98%, Mn 17012) was supplied by Tianjin Fuchen Chemical Reagent Co., Ltd., Tianjin, China. All the reagents above were analytical grade. PEG was dehydrated under reduced pressure at 80 °C for 3 h. TA was dried in a vacuum oven at 60 °C for 3 h before use. IPDI was used as received.

### 2.2. Sample Preparation

The ultra-high-filled wood flour dynamic polyurethane (UWPU) composites containing 60–90% wood flour content were prepared via in situ polymerization and a hot-pressed process. The formulations of UWPU composites are shown in Table 1. As a representative procedure, the UWPU-80 was synthesized as follows: 6 g of PEG and 0.68 g of TA were dissolved in 5 mL of DMF. Then, 3.3 g of IPDI (the molar ratio of hydroxyl and isocyanate was 1:1) was added to the mixture and uniformly stirred at room temperature. Afterward, poplar wood flour (40 g) was added to the mixture and stirred until the mixture became homogenized. Then, DMF was evaporated by heating the sample at 80 °C for 2 h in a vacuum-drying oven to obtain poplar wood flour particles wrapped by a dynamic polyurethane network. Finally, the UWPU-80 composite was obtained by hot-pressing the sample at 10 MPa and 120 °C for 2 h.

### 2.3. Analysis

#### 2.3.1. Fourier-Transform Infrared (FT-IR) Spectroscopy Analysis

The FT-IR spectra were recorded using an attenuated total reflectance spectrometer (Nicolet 6700, Thermo Fisher Scientific, Waltham, MA, USA). The spectra were recorded with a wave number range of 600–4000 cm^−1^ with 32 times with a 4 cm^−1^ resolution.

#### 2.3.2. Scanning Electron Microscope (SEM) Analysis

The morphology of the UWPU composites was examined using an electronic scanning microscope (SEM, JEM-2100). The UWPU composites were carefully mounted on the aluminum stubs, sputter-coated with 20 nm gold for conductivity at an accelerating voltage of 12.5 kV.

#### 2.3.3. Dynamic Thermomechanical Analysis (DMA)

DMA was performed using a dynamic mechanical analyzer (TA-Q800) in single cantilever beam mode. All samples measuring 35 mm × 12 mm × 3 mm were scanned from room temperature to 150 °C, except for UWPU-80, which was scanned from −50 to 150 °C. The measurements were conducted at a heating rate of 3 °C/min, an amplitude of 15 µm, and a frequency of 1 Hz.

#### 2.3.4. Creep Analysis

A dynamic mechanical analyzer (TA-Q800) was used to test the short-term creep performance of the composite material. The creep mode was selected, and each specimen was 35 mm × 12 mm × 3 mm. The test fixture was selected as a single cantilever, and a stress of 0.5 MPa was applied at isothermal conditions with a loading time of 30 min and a releasing time of 30 min. Isothermal creep tests were performed at 30 and 60 °C.

#### 2.3.5. Thermomechanical Analysis (TMA)

The thermal expansion of the composites was measured using a thermomechanical analyzer (Q400; TA Instruments Inc., New Castle, DE, USA). The samples (6 mm × 6 mm × 4 mm) were clamped between the probe and the sample table at a force of 0.05 N. Then, they were heated from 30 to 60 °C, cooled to −30 °C, and finally reheated to 30 °C. The heating and cooling rates remained the same at 3 °C/min.

#### 2.3.6. Mechanical Properties

The flexural, tensile, and unnotched impact tests were measured according to ASTMD 790–10, ASTM D638, and ASTM D6110-2017 standards, respectively. The flexural and tensile properties of the UWPU composites were measured at room temperature using a universal mechanical testing machine (CMT5504, MTS Systems Co., Ltd., Shanghai, China). Flexural property tests were conducted on samples with dimensions of 80 mm × 13 mm × 4 mm, whereas tensile property tests were performed on the samples with dimensions of 80 mm × 10 mm × 4 mm.

Each mechanical test was repeated seven times. The flexural and tensile properties of the samples were measured at a speed of 2 mm/min. The unnotched Izod impact strength of the composites was measured using an impact testing machine with 5 ft-lb pendulums (JC5, Chengde Jingmi Testing Machine Co., Ltd., Chengde, China). Samples with dimensions of 80 mm × 10 mm × 4 mm were used for the unnotched Izod impact strength.

#### 2.3.7. Recycling Test

Two testing methods were used for this study: mechanical handling and chemical dissolution reprocessing tests. In the mechanical handling reprocessing test, a sample of UWPU-80 composite was ground into powders and molded into recycled UWPU-80 composite by hot-pressing the sample at 10 MPa and 120 °C for 2 h. In the chemical dissolution reprocessing test, the UWPU-80 composite sample was degraded using PEG600. TA and IPDI monomers were added to the mixture in the original ratio without separating the mixture. After 80% wood flour was added to the mixture, a new batch of composite material was formed by hot-pressing the sample according to the sample preparation method.

## 3. Results

### 3.1. Preparation of UWPU Composites

**Figure 1:**
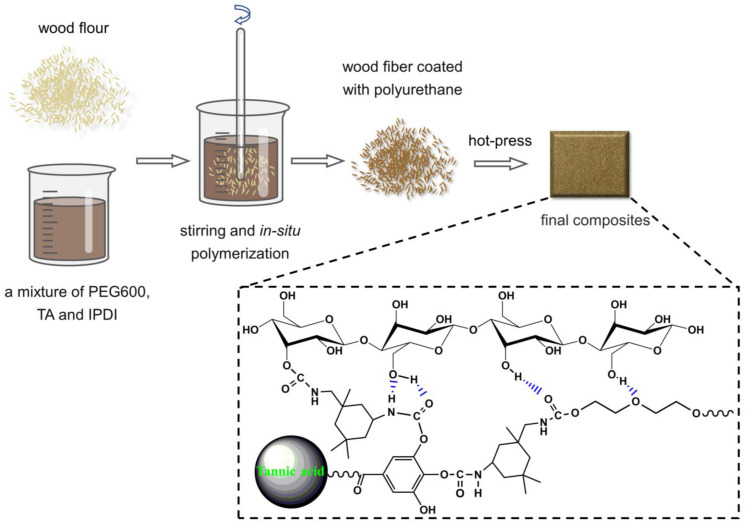
Schematic representation of the preparation of the UWPU composites.

Figure 1 illustrates the preparation process of UWPU composites. Dynamic polyurethanes were in situ polymerized on the surface of wood fibers in a solvent. Then, the polymer was tightly cross-linked with the biomass via a simple hot-press molding process. The phenol–carbamate bonds in dynamic polyurethanes break down into phenol hydroxy groups and isocyanate groups at 120 °C. When cooled to room temperature, the phenol–carbamate bonds recombine with phenolic hydroxyl groups on TA and isocyanate groups, while some isocyanate groups may react with hydroxyl groups on wood fibers. Both the polyurethane and wood fiber contain a large number of polar groups, thus, hydrogen bonding may exist between the hydroxy groups of the wood fiber and the polyurethane’s phenol–carbamate and ester groups [35,36]. These hydrogen bonding interactions may improve the compatibility of polyurethane and wood fiber significantly.

**Figure 2:**
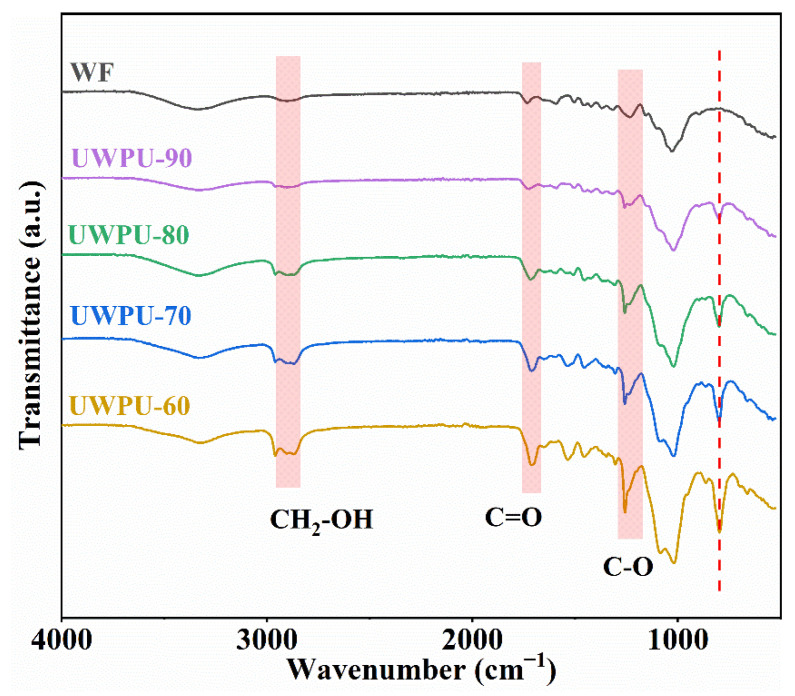
FT-IR spectra of UWPU composites.

The FT-IR absorption spectra of the UWPU composites reveal the interaction between the carbamate bonds in the polyurethane and the hydroxyl groups on the surface of the wood fibers (Figure 2). The absorption peak at 2903 cm^−1^ is considered to be the symmetric stretching vibration absorption peak of CH_2_ in CH_2_-OH, and in the composite, the peak splits into two at 2960 cm^−1^ and 2869 cm^−1^, representing the asymmetric and symmetric stretching vibration absorption peaks of CH_2_ in polyurethane, respectively, both of which decrease in intensity with increasing wood flour content [37,38]. The absorption peak at 1734–1712 cm^−1^ corresponds with the stretching vibration of C=O, and the peak increases with increasing polymer content. The absorption peak at 1018–1258 cm^−1^ corresponds with the C-O vibration absorption peak, which also increases with increasing polymer content. Analysis of the infrared spectrograms reveal the characteristic peak of TA (799 cm^−1^) in all composites. The intensity of the peak increases with increasing content of TA [39,40]. As the dynamic phenol–carbamate bonds in the polymer separate during the hot-pressing process, some of the tannic acid is freed. Thereby, it can be speculated that the isocyanate in the polymer may form covalent bonds with the hydroxyl groups on the wood fiber. These characteristic absorption peaks confirm that the dynamic polyurethane polymer is successfully coated on the wood fibers [41].

### 3.2. Thermal and Mechanical Properties

**Figure 3:**
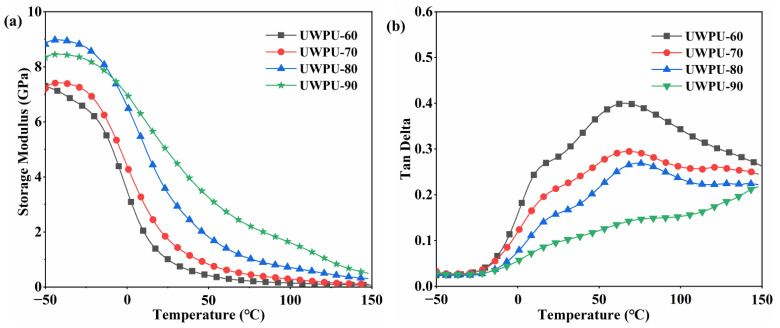
(**a**) Storage modulus and (**b**) tan δ curves of UWPU composites.

Dynamic mechanical analysis can be used to obtain information about the thermoset structure of biomass composites. The storage modulus reflects the stiffness of the material, and the wood flour has strong stiffness. When the filling amount of wood flour is higher, the composite is stiffer [12]. Figure 3a shows the storage modulus of UWPU composites with different wood fiber fillings. At approximately −15 °C, the storage modulus of the composites rapidly decreases. At room temperature, the storage modulus increases with increasing wood fiber content. The storage modulus of UWPU-60, UWPU-70, and UWPU-80 gradually level off after 100 °C. Figure 3b shows the temperature dependence of the mechanical loss factor tan δ of UWPU composites containing different wood fiber contents. The peak temperature of the tan-T curve is the glass transition temperature (T_g_) of UWPU. As the wood fiber content increases, the intensity of tan δ peaks decrease and show a positive shift. Wood flour has a limiting effect on the chain segment motion of the polymer, which is enhanced with increasing wood flour content. Wood fibers can be considered as macro-crosslinkers of UWPU. When the wood fiber contents increase, the overall cross-linkage of the composite increase, thus, limiting the molecular movement of the composite. A shoulder seam is observed within the range of 10–20 °C for each curve. This phenomenon could be attributed to the T_g_ of the polymer that is not tightly bound to the wood fiber surface. The temperatures corresponding to the shoulder peaks of UWPU-60, UWPU-70, and UWPU-80 are 12.88, 15.36, and 18.37 °C, respectively, which show the same trend as the highest peak of the composite. The trend of the lower T_g_ indicates an increase in the mobility of the polymer chains. Wood fibers can limit the migration of polymer chains by forming hydrogen bonds and covalent bonds in the interface, thus, interrupting the interactions between the matrix polymer chains [5,12,42]. The lower T_g_ also indicates a good interfacial adhesion between the wood fibers and the polymer. The lower T_g_ in UWPU-90 is not obvious because the content of dynamic polyurethane is relatively low in UWPU-90.

**Figure 4:**
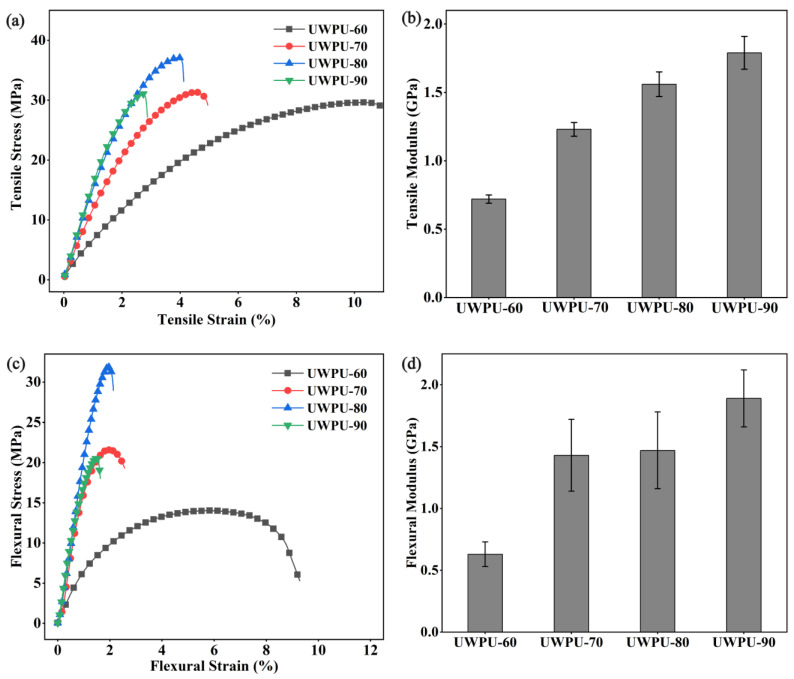
Mechanical properties of UWPU composites: (**a**) tensile stress–strain curves; (**b**) Young’s modulus; (**c**) three-point flexural stress–strain curves; (**d**) flexural modulus.

**Figure 5:**
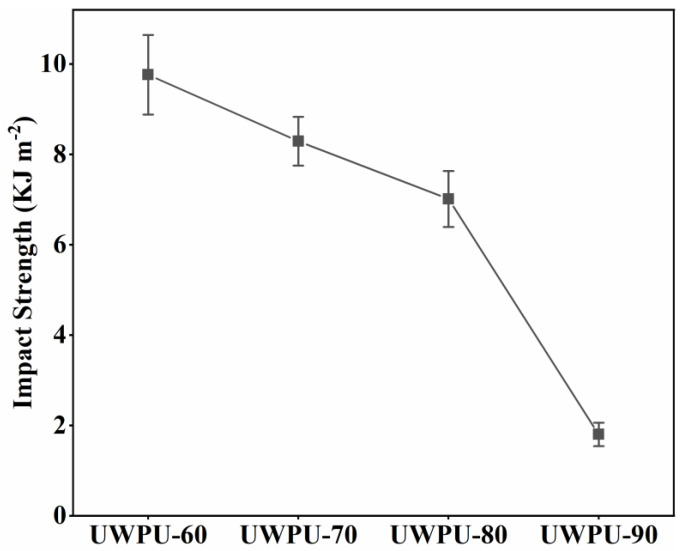
Unnotched Izod impact strength of UWPU composites.

**Figure 6:**
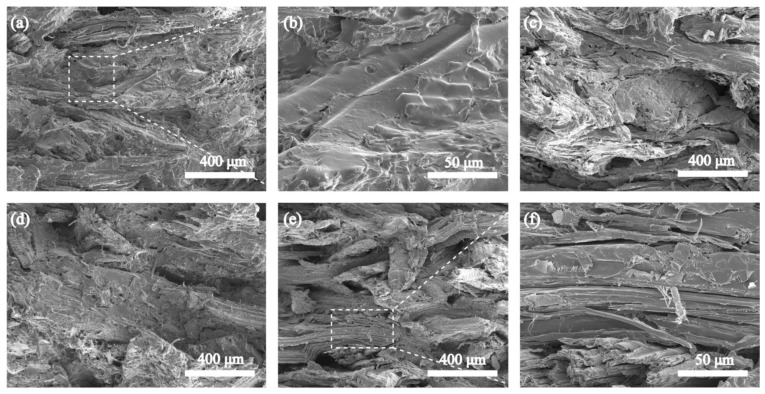
The SEM images of UWPU-60 (**a**,**b**), UWPU-70 (**c**), UWPU-80 (**d**), and UWPU-90 (**e**,**f**).

Figure 4a–d and Figure 5 show the tensile, three-point bending, and unnotched impact tests of UWPU composites, respectively. The tensile strength and flexural strength of UWPU composites initially increase and then decrease with the increase in wood flour content. The maximum tensile and flexural strength are 37 and 33 MPa, respectively, obtained from the composite with 80% wood flour content. The improved mechanical properties of the composites are attributed to the reinforcing effect of the wood fibers. The poor performance of UWPU-90 is due to the defects inside the composite material caused by the high filling of wood flour. These defects generate stress concentration when the sample is subjected to external forces, resulting in poor mechanical properties. The SEM images of the tensile sections of the composites show that the sections became pitted from flat as the amount of wood flour filling increases (Figure 6). This phenomenon is because polyurethane contains a large number of polar groups that are compatible with wood flour, which has polarity. The cross-linking of phenol–carbamate bonds with hydroxyl groups on wood fibers after thermal depolymerization also improves their compatibility with wood flour. However, when the wood flour content increases to 90%, more holes are left in the tensile section where the wood fibers are pulled out (Figure 6e,f), which is due to there being insufficient polymer to completely coat the wood fibers at the high wood flour content. The tensile and flexural modulus of the UWPU composites gradually increase with increased wood flour content. Generally, the modulus reflects the stiffness of the material. Thus, the stiffness of the wood flour increases the overall stiffness of the composite with increasing wood flour content. The unnotched impact performance of the UWPU composites gradually decrease with increasing wood flour content (Figure 5). Wood fibers in composites are encapsulated by flexible polymers, which inhibit crack generation and propagation during impact damage and absorb more energy when damaged, showing higher impact toughness [43]. In addition, the defects within the composite material decrease impact strength. 

### 3.3. Creep Property

**Figure 7:**
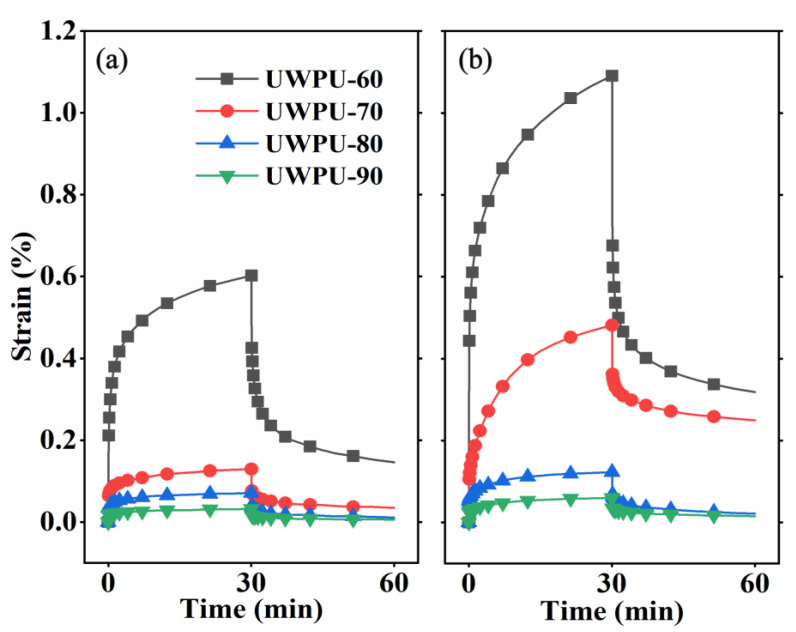
Creep curves of UWPU composites at (**a**) 30 °C and (**b**) 60 °C.

The prepared UWPU composites contain dynamic polyurethane polymer that is liable to creep, which can greatly affect the use of the material. The creep of the polymer includes the superposition of recoverable and non-recoverable deformation. The creep strain of UWPU-60 is about 0.6% after 30 min at 30 °C. Under the same conditions, the creep strain is about 0.02% when the wood flour content is 90%, which decreases by approximately 96.7%, indicating that the creep gradually decreases with increasing wood flour content (Figure 7a). The improved creep resistance in UWPU composites is mainly attributed to the content of rigid wood flour, which restricts the movement of polymer molecular chain segments, thus, reducing creep deformation [44,45]. The residual and non-recoverable deformations display a similar trend to creep deformation with increasing wood flour content. The chain segment motion of dynamic polyurethane polymers increases at 60 °C, resulting in more creep strain and residual deformation. The creep strain and residual deformations of UWPU at 60 °C are about twice as high than those at 30 °C (Figure 7b). Under higher temperature conditions, the high filling amount of wood flour exhibits more significant anti-creep properties, and the residual deformation is slightly different in the composites with 80% and 90% wood flour content. The incorporation of 80% wood flour can effectively reduce the creep deformation of the composites.

### 3.4. Thermal Expansion Property

In composites, the LCTE is an essential property; a low LCTE value is more favorable for the dimensional stability of the composite [44,46]. The LCTE of the composite in the thickness direction was tested at three temperature stages (Table 2). At each temperature stage, the LCTE of the UWPU composites decrease with increasing wood flour content. The decrease in the LCTE of UWPU composites indicates that the wood fibers play an inhibitory role in the UWPU composites, and the thermal expansion behavior of the composites is mainly due to the polyurethane matrix. During the heating or cooling cycle, the wood fibers mechanically constrain the movement of the polymer chain segments, which effectively reduces the LCTE value of the composite.

### 3.5. Recycling Ability

**Figure 8:**
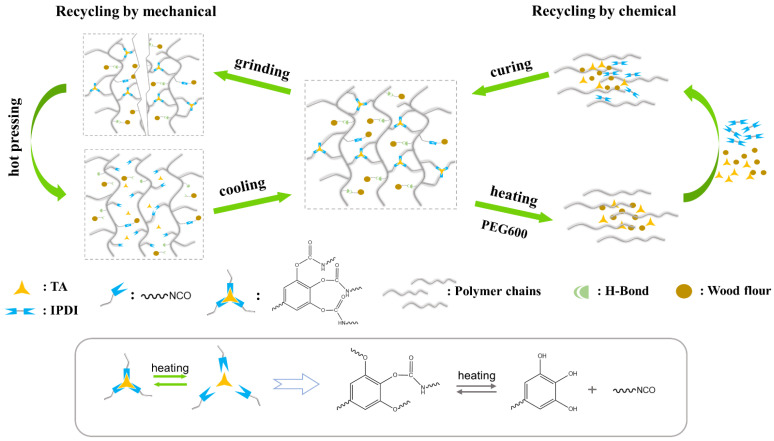
Schematic of the recycling mechanism of UWPU composites.

**Figure 9:**
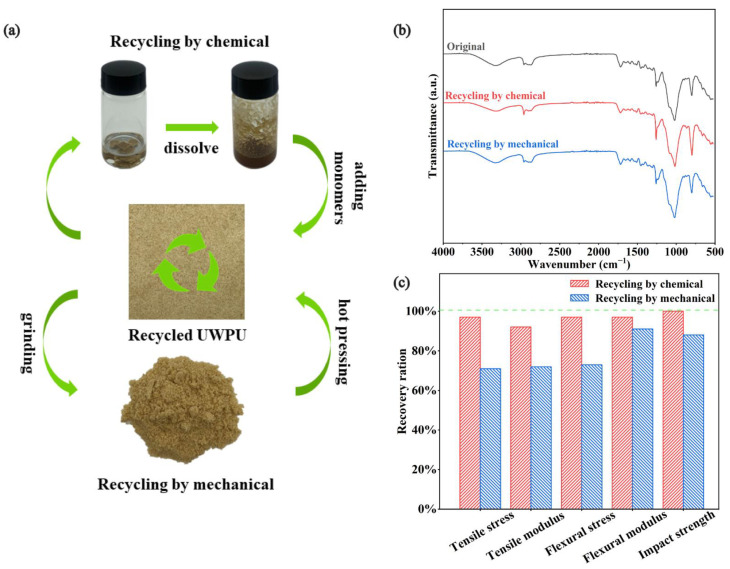
Recycling properties of the UWPU: (**a**) the digital photographs of the chemical recycling process and the mechanical recycling process, (**b**) the FT-IR spectra of the recycled UWPU, and (**c**) the recovery ratio of tensile strength, tensile modulus, flexural strength, bending modulus, and impact strength of the recycled UWPU.

**Figure 10:**
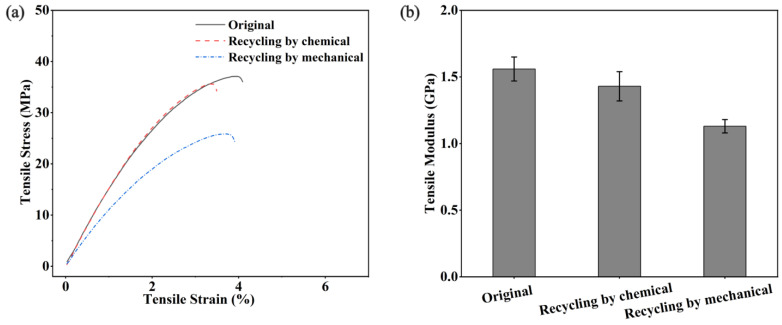

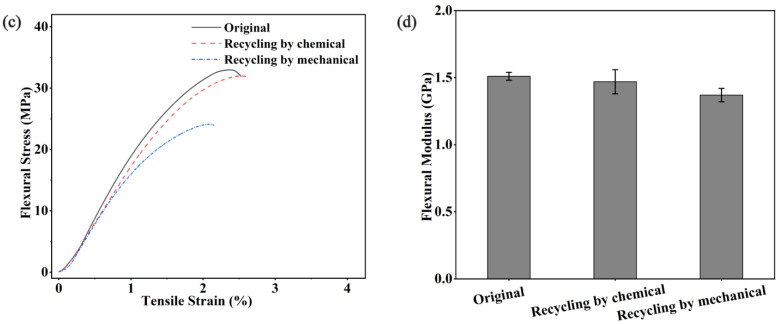
Mechanical properties of the original and recycled UWPU composites: (**a**) tensile stress–strain curves; (**b**) tensile modulus; (**c**) three-point flexural stress–strain curves; (**d**) flexural modulus.

Biodegradable and recyclable materials align with the circular bioeconomy. In addition, they are more environmentally friendly and have low carbon [47,48]. In this study, we used two recovery methods: chemical and mechanical recoveries (Figure 8 and Figure 9a). Polyurethanes cross-linked by dynamic chemical bonds can be depolymerized by introducing excess hydroxyl groups to disrupt the stoichiometric balance between phenolic hydroxyl and isocyanate groups in the network [34]. UWPU-80 is fully degraded when UWPU-80 is immersed in a PEG solution and then heated and stirred at 120 °C for 3 h. In the same procedure, a closed-loop recycled composite is obtained by adding wood flour and a certain stoichiometric ratio of TA and IPDI monomers to the recycled solution, where the molar ratio of -NCO from IPDI to -OH from PEG and TA is 1:1, followed by hot pressing. Moreover, the recovered composites have almost the same mechanical properties as the original specimens. The tensile strength, tensile modulus, flexural strength, bending modulus, and impact strength of the recovered composites are 97, 92, 97, 97, and 100%, respectively, of those of the original specimens (Figure 9c and Figure 10). As shown in Figure 9b, the recovered UWPU-80 has the same chemical structure as the original UWPU-80. In addition, the dynamic polyurethane is depolymerized under heat. The stress relaxation ability of the urethane bond in the dynamic polyurethane activated by heat causes interfacial healing between the wrapped wood flour [49]. For mechanical recycling, UWPU-80 is ground into powder and re-press molded. The mechanically recycled UWPU-80 has excellent mechanical property recovery (tensile strength, tensile modulus, flexural strength, bending modulus, and impact strength recoveries of 71, 72, 73, 91, and 88%, respectively) (Figure 9c and Figure 10). In addition, the chemical structure of mechanically recycled UWPU-80 is similar to that of the original UWPU-80 (Figure 9b).

## 4. Conclusions

In this work, composite materials containing 60–90 wt% biomass were prepared using dynamic polyurethane as a matrix, and the closed-loop recycling properties of composite materials were investigated. The thermal and mechanical properties of the UWPU were adjusted by changing the wood flour content. FTIR, SEM, and DMA measurements reveal good compatibility between the polyurethane and wood flour in the composites when the wood flour content is ≤80 wt%. The mechanical properties of the composites initially increase and then decrease. The maximum tensile and bending strength of the composite are 37 and 33 MPa when the wood flour content is 80%. The composites with higher wood flour content demonstrate higher thermal expansion stability and creep resistance. Due to the introduction of dynamic phenol–carbamate bonds in the composites, the UWPU composites were mechanically crushed and maintain good mechanical properties and chemical structure after reprocessing. The UWPU composites are depolymerized into wood flour and hydroxyl-capped polyurethane prepolymers with the assistance of heat when there is a surplus of hydroxyl groups in the solvent. Then, the depolymerized wood flour and the polymers are remolded by simply supplementing them with other monomers. The recovered composite has a 97% recovery in tensile and flexural strength and retains the chemical structure of the original composite. This work used a simple preparation process and environmentally friendly recycling methods to achieve full reprocessing of materials. This method can be used for establishing a green, low-carbon, circular development economic system. The recyclable composites prepared in this work have great potential for applications in smart furniture, the construction industry, and automotive interior manufacturing.

## Figures and Tables

**Table 1:** Formulations of preparing UWPU composites.

Sample	PEG (g)	TA (g)	IPDI (g)	Wood Flour (g)	Wood Flour Content (wt%)
UWPU-60	6.00	0.68	3.33	15.01	60
UWPU-70	6.00	0.68	3.33	23.37	70
UWPU-80	6.00	0.68	3.33	40.04	80
UWPU-90	6.00	0.68	3.33	90.09	90

**Table 2:** Linear coefficient of thermal expansion (LCTE) of the UWPU composites measured in the thickness direction at temperatures changing from 30 to 60 °C, 60 to −30 °C, and −30 to 30 °C.

Samples	Single LCTE (10^−6^ °C^−1^)		
	30→60	60→(−30)	(−30)→30
UWPU-60	125.75	184.02	136.74
UWPU-70	93.91	162.65	121.85
UWPU-80	63.71	136.37	96.64
UWPU-90	51.82	115.83	89.25

## Data Availability

Not applicable.

## References

[1] Guan Q.F., Yang H.B., Han Z.M., Ling Z.C., Yu S.H. (2020). An all-natural bioinspired structural material for plastic replacement. Nat. Commun..

[2] Guillaume S.M. (2022). Sustainable and degradable plastics. Nat. Chem..

[3] Mukherjee T., Czaka M., Kao N., Gupta R.K., Choi H.J., Bhattacharya S. (2014). Dispersion study of nanofibrillated cellulose based poly(butylene adipate-co-terephthalate) composites. Carbohydr. Polym..

[4] Chan C.M., Vandi L.-J., Pratt S., Halley P., Richardson D., Werker A., Laycock B. (2018). Mechanical performance and long-term indoor stability of polyhydroxyalkanoate (PHA)-based wood plastic composites (WPCs) modified by non-reactive additives. Eur. Polym. J..

[5] Nair S.S., Chen H., Peng Y., Huang Y., Yan N. (2018). Polylactic Acid Biocomposites Reinforced with Nanocellulose Fibrils with High Lignin Content for Improved Mechanical, Thermal, and Barrier Properties. ACS Sustain. Chem. Eng..

[6] Sommerhuber P.F., Wenker J.L., Rüter S., Krause A. (2017). Life cycle assessment of wood-plastic composites: Analysing alternative materials and identifying an environmental sound end-of-life option. Resour. Conserv. Recycl..

[7] Ou R., Xie Y., Wang Q., Sui S., Wolcott M.P. (2014). Effects of ionic liquid on the rheological properties of wood flour/high density polyethylene composites. Compos. Part A Appl. Sci. Manuf..

[8] Xie Y., Hill C.A.S., Xiao Z., Militz H., Mai C. (2010). Silane coupling agents used for natural fiber/polymer composites: A review. Compos. Part A Appl. Sci. Manuf..

[9] Ou R., Wang Q., Wolcott M.P., Sui S., Xie Y., Song Y. (2014). Effects of chemical modification of wood flour on the rheological properties of high-density polyethylene blends. J. Appl. Polym. Sci..

[10] Yuan L., Buzoglu Kurnaz L., Tang C. (2021). Alternative plastics. Nat. Sustain..

[11] Oliaei E., Olsen P., Lindstrom T., Berglund L.A. (2022). Highly reinforced and degradable lignocellulose biocomposites by polymerization of new polyester oligomers. Nat. Commun..

[12] Subbotina E., Montanari C., Olsén P., Berglund L.A. (2022). Fully bio-based cellulose nanofiber/epoxy composites with both sustainable production and selective matrix deconstruction towards infinite fiber recycling systems. J. Mater. Chem. A.

[13] Liu Y., Yu Z., Wang B., Li P., Zhu J., Ma S. (2022). Closed-loop chemical recycling of thermosetting polymers and their applications: A review. Green Chem..

[14] Saitta L., Prasad V., Tosto C., Murphy N., Ivankovic A., Cicala G., Scarselli G. (2022). Characterization of biobased epoxy resins to manufacture eco-composites showing recycling properties. Polym. Compos..

[15] Kumar S., Krishnan S. (2020). Recycling of carbon fiber with epoxy composites by chemical recycling for future perspective: A review. Chem. Pap..

[16] Palmer J., Ghita O.R., Savage L., Evans K.E. (2009). Successful closed-loop recycling of thermoset composites. Compos. Part A Appl. Sci. Manuf..

[17] Roy N., Bruchmann B., Lehn J.M. (2015). DYNAMERS: Dynamic polymers as self-healing materials. Chem. Soc. Rev..

[18] Chakma P., Konkolewicz D. (2019). Dynamic Covalent Bonds in Polymeric Materials. Angew. Chem. Int. Ed. Engl..

[19] Wojtecki R.J., Meador M.A., Rowan S.J. (2011). Using the dynamic bond to access macroscopically responsive structurally dynamic polymers. Nat. Mater..

[20] Denissen W., Winne J.M., Du Prez F.E. (2016). Vitrimers: Permanent organic networks with glass-like fluidity. Chem. Sci..

[21] Van Zee N.J., Nicolaÿ R. (2020). Vitrimers: Permanently crosslinked polymers with dynamic network topology. Prog. Polym. Sci..

[22] Wu H., Liu X., Sheng D., Zhou Y., Xu S., Xie H., Tian X., Sun Y., Shi B., Yang Y. (2021). High performance and near body temperature induced self-healing thermoplastic polyurethane based on dynamic disulfide and hydrogen bonds. Polymer.

[23] Suzuki N., Takahashi A., Ohishi T., Goseki R., Otsuka H. (2018). Enhancement of the stimuli-responsiveness and photo-stability of dynamic diselenide bonds and diselenide-containing polymers by neighboring aromatic groups. Polymer.

[24] Du W., Jin Y., Pan J., Fan W., Lai S., Sun X. (2018). Thermal induced shape-memory and self-healing of segmented polyurethane containing diselenide bonds. J. Appl. Polym. Sci..

[25] Cash J.J., Kubo T., Dobbins D.J., Sumerlin B.S. (2018). Maximizing the symbiosis of static and dynamic bonds in self-healing boronic ester networks. Polym. Chem..

[26] Zhao Z.-H., Wang D.-P., Zuo J.-L., Li C.-H. (2021). A Tough and Self-Healing Polymer Enabled by Promoting Bond Exchange in Boronic Esters with Neighboring Hydroxyl Groups. ACS Mater. Lett..

[27] Zhang J., Zhang C., Song F., Shang Q., Hu Y., Jia P., Liu C., Hu L., Zhu G., Huang J. (2022). Castor-oil-based, robust, self-healing, shape memory, and reprocessable polymers enabled by dynamic hindered urea bonds and hydrogen bonds. Chem. Eng. J..

[28] Zechel S., Geitner R., Abend M., Siegmann M., Enke M., Kuhl N., Klein M., Vitz J., Gräfe S., Dietzek B. (2017). Intrinsic self-healing polymers with a high E-modulus based on dynamic reversible urea bonds. NPG Asia Mater..

[29] Wu P., Cheng H., Wang X., Shi R., Zhang C., Arai M., Zhao F. (2021). A self-healing and recyclable polyurethane-urea Diels–Alder adduct synthesized from carbon dioxide and furfuryl amine. Green Chem..

[30] Ehrhardt D., Van Durme K., Jansen J.F.G.A., Van Mele B., Van den Brande N. (2020). Self-healing UV-curable polymer network with reversible Diels-Alder bonds for applications in ambient conditions. Polymer.

[31] Liu Y., Zhang Z., Fan W., Yang K., Li Z. (2022). Preparation of renewable gallic acid-based self-healing waterborne polyurethane with dynamic phenol–carbamate network: Toward superior mechanical properties and shape memory function. J. Mater. Sci..

[32] Shi J., Zheng T., Zhang Y., Guo B., Xu J. (2019). Reprocessable Cross-Linked Polyurethane with Dynamic and Tunable Phenol–Carbamate Network. ACS Sustain. Chem. Eng..

[33] Shi J., Zheng T., Guo B., Xu J. (2019). Solvent-free thermo-reversible and self-healable crosslinked polyurethane with dynamic covalent networks based on phenol-carbamate bonds. Polymer.

[34] Cao S., Li S., Li M., Xu L., Ding H., Xia J., Zhang M., Huang K. (2017). A thermal self-healing polyurethane thermoset based on phenolic urethane. Polym. J..

[35] Jin Y., Lei Z., Taynton P., Huang S., Zhang W. (2019). Malleable and Recyclable Thermosets: The Next Generation of Plastics. Matter.

[36] Su Z., Hu Y., Yang X., Long R., Jin Y., Wang X., Zhang W. (2020). Production and closed-loop recycling of biomass-based malleable materials. Sci. China Mater..

[37] Schwanninger M., Rodrigues J.C., Pereira H., Hinterstoisser B. (2004). Effects of short-time vibratory ball milling on the shape of FT-IR spectra of wood and cellulose. Vib. Spectrosc..

[38] Chen L., Wang X., Yang H., Lu Q., Li D., Yang Q., Chen H. (2015). Study on pyrolysis behaviors of non-woody lignins with TG-FTIR and Py-GC/MS. J. Anal. Appl. Pyrolysis.

[39] Luo J., Lai J., Zhang N., Liu Y., Liu R., Liu X. (2016). Tannic Acid Induced Self-Assembly of Three-Dimensional Graphene with Good Adsorption and Antibacterial Properties. ACS Sustain. Chem. Eng..

[40] Lin Q., Wu J., Yu Y., Huang Y., Yu W. (2020). Immobilization of ferric tannate on wood fibers to functionalize wood fibers/diphenylmethane di-isocyanate composites. Ind. Crops Prod..

[41] Fan H., Wang L., Feng X., Bu Y., Wu D., Jin Z. (2017). Supramolecular Hydrogel Formation Based on Tannic Acid. Macromolecules.

[42] Liu H., Liu D., Yao F., Wu Q. (2010). Fabrication and properties of transparent polymethylmethacrylate/cellulose nanocrystals composites. Bioresour. Technol..

[43] Huang R., Zhang X., Zhou C. (2020). Mechanical, flammable, and thermal performances of co-extruded wood polymer composites with core–shell structure containing barite-filled shells. Wood Sci. Technol..

[44] Hao X., Zhou H., Xie Y., Mu H., Wang Q. (2018). Sandwich-structured wood flour/HDPE composite panels: Reinforcement using a linear low-density polyethylene core layer. Constr. Build. Mater..

[45] Jia M., Xue P., Zhao Y., Wang K. (2009). Creep behaviour of wood flour/poly(vinyl chloride) composites. J. Wuhan Univ. Technol.-Mater. Sci. Ed..

[46] Wu Q., Chi K., Wu Y., Lee S. (2014). Mechanical, thermal expansion, and flammability properties of co-extruded wood polymer composites with basalt fiber reinforced shells. Mater. Des..

[47] Al-Etaibi A.M., El-Apasery M.A., Mahmoud H.M., Al-Awadi N.A. (2012). One-Pot Synthesis of Disperse Dyes Under Microwave Irradiation: Dyebath Reuse in Dyeing of Polyester Fabrics. Molecules.

[48] Al-Etaibi A.M., El-Apasery M.A. (2022). Facile Synthesis of Novel Disperse Dyes for Dyeing Polyester Fabrics: Demonstrating Their Potential Biological Activities. Polymers.

[49] Whiteley J.M., Taynton P., Zhang W., Lee S.H. (2015). Ultra-thin Solid-State Li-Ion Electrolyte Membrane Facilitated by a Self-Healing Polymer Matrix. Adv. Mater..

